# Chemotherapy Combined With Surgery in a Case With Metanephric Adenoma

**DOI:** 10.3389/fped.2022.847864

**Published:** 2022-04-07

**Authors:** Shaohua Hu, Zhenli Zhao, Zhisheng Wan, Weizhen Bu, Songqiang Chen, Yiqun Lu

**Affiliations:** ^1^Department of Urology, Hainan Women and Children’s Medical Center, Haikou, China; ^2^Department of Urology, Children’s Hospital of Fudan University, Shanghai, China

**Keywords:** metanephric adenoma, children, chemotherapeutics, operation treatment, nephron sparing surgery

## Abstract

**Background:**

Metanephric adenoma is an extremely rare renal neoplasm, especially in pediatrics. Chemotherapy combined with surgery in metanephric adenoma has not been reported.

**Methods:**

We describe a case of metanephric adenoma in a child less than 2 years old, which were treated by chemotherapy combined with surgery.

**Results:**

Nephron sparing surgery was performed after regular chemotherapy, and the pathological result was metanephric adenoma.

**Conclusion:**

Pediatric metanephric adenoma is extremely rare; the clinical manifestations and imaging examinations lack specificity. Nephron sparing surgery is recommended as the preferred treatment for metanephric adenoma. Long-term follow-up and more in-depth molecular genetic research are still needed to determine the benign or malignant of metanephric adenoma and whether chemotherapy drugs have an effect on it.

## Introduction

Metanephric adenoma (MA) is an extremely rare epithelial tumor of the kidney, which accounts for about 0.2% of all renal epithelium-derived tumors. This neoplasm mostly occurs in patients aged 50–60 years old, with a female-to-male ratio of 2:1 (1, 2). Since Bove et al. (3) first discovered MA in 1979, only a few hundred cases have been reported, and most of them are adults. The incidence of MA in children is extremely rare, with dozens of cases reported at present, and only less than five cases in children under 2 years old (4–7). We reported a child less than 2 years old with postoperative pathological confirmed MA, who was considered as nephroblastoma by preoperative examination and received chemotherapy before surgical treatment. To our knowledge, this is the first case of a child with MA receiving a chemotherapeutic agent. The summary is as follows to improve clinicians’ understanding of MA.

## Materials and Methods

### Subjects and Ethical Approval

**FIGURE 1:**
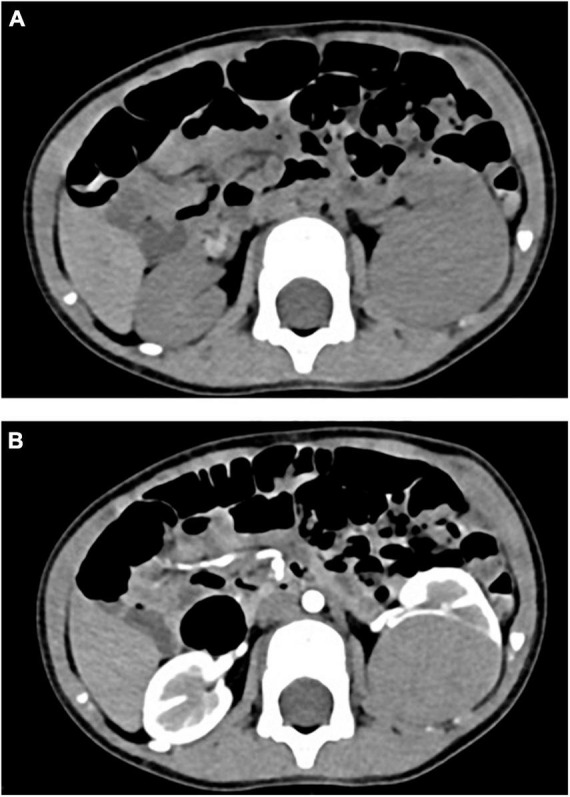
**(A)** CT plain scan before chemotherapy: CT value was 46 HU. **(B)** CT enhanced before chemotherapy: the third stage showed obvious uneven enhancement, with CT values of about 58 HU, 98 HU, and 133 HU, respectively.

**FIGURE 2:**
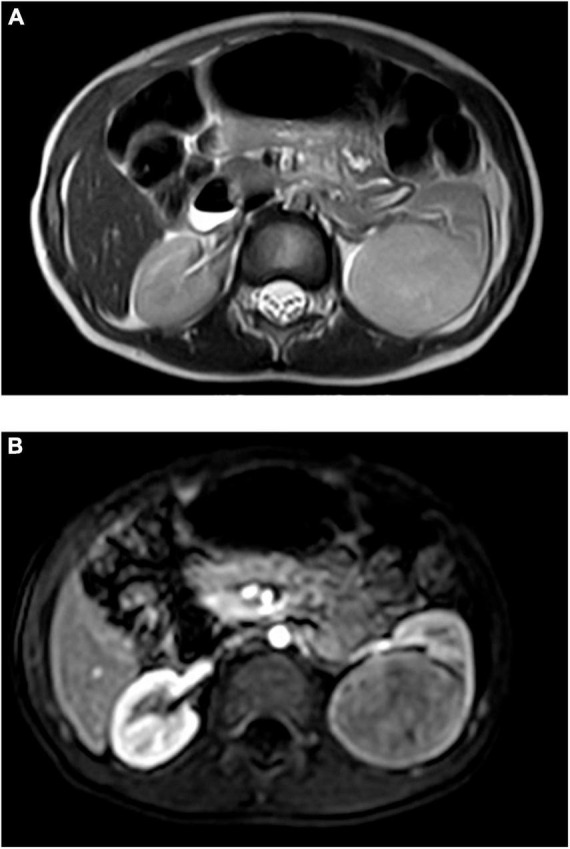
**(A)** MRI plain scan before chemotherapy: equisignal on T1WI, slightly higher signal on T2WI, significantly higher signal on DWI, and low signal on ADC. **(B)** MRI enhancement before chemotherapy: dynamic enhancement showed delayed uneven enhancement, and the degree was weaker than that of renal cortex.

**FIGURE 3:**
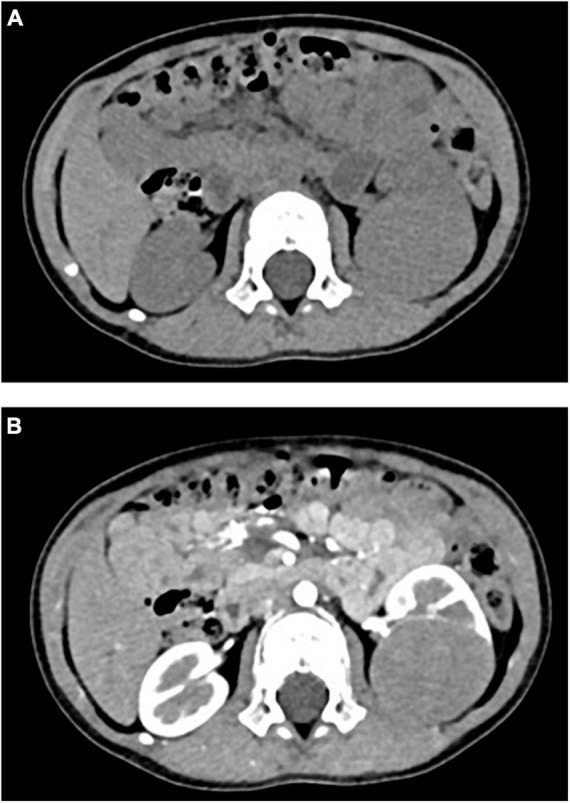
**(A)** CT plain scan after chemotherapy: CT value was 43HU. **(B)** CT enhanced after chemotherapy: the third stage showed obvious uneven enhancement, with CT values of about 63HU, 101HU, and 135HU, respectively.

Retrospective analysis was performed on the case data of a child with MA admitted to the Department of Urology, Hainan Women and Children’s Medical Center, on 13 September 2021. The child aged 1 year and 11 months was accidentally examined by urologic color Doppler ultrasonography in a local hospital, and found to have a hypoechoic mass in the left kidney. He was then admitted to our hospital for further treatment. Physical examination showed no abnormality. Color ultrasound showed that a substantial mass with a size of 54 × 43 × 44 mm could be detected in the middle and lower pole of the left kidney, which was surrounded by a capsule. The mass was protruding out of the capsule with clear boundary, and mainly parenchymal echo; a small number of no echo areas also could be seen in it. The nodular growth of the mass was observed without obvious strong echogenic. CT suggested a solid space-occupying lesion in the middle and lower segment of the left kidney, about 36.70 × 42.10 × 50.60 mm in size, and nephroblastoma (stage I) was considered (Figure 1). MRI reviewed a solid space occupying lesion in the lower pole of the left kidney, and the possibility of nephroblastoma was considered (Figure 2). CT reexamination after chemotherapy showed that the solid mass was smaller than before, and the maximum cross-sectional size was about 36.16 × 39.27 × 49.65 mm (Figure 3). Neuron-specific enolase (NSE) assay was 25.71 ng/ml (reference value: 0.00–16.30 ng/ml), urine vanillylmandelic acid (VMA), Hb: 139 g/L (reference range: 105–145 g/L), and MCV: 79.9 fL (reference range: 80–100 fL); blood routine examination showed no abnormality.

This study was approved by the Review Board of the Hainan Women and Children’s Medical Center, Haikou, China. Written informed consent was obtained from the legal guardian of the child.

### Methods

#### Preoperative Chemotherapy

According to CCCG-WT-2016 (8), the child was treated with EE4A chemotherapy.

#### Surgical Method

The left trans-costal incision was made after completing preoperative preparation. The tumor was located in the middle and inferior pole of the left kidney and protruded on the renal surface; the capsule was intact, the boundary with the renal parenchyma was clear, and it was not connected with the renal binding system. There were no enlarged lymph nodes in the retroperitoneum, renal hilum, and abdominal aorta. The tumor nutrient artery came from the left renal artery. Partial left nephrectomy plus left perirenal lymph node dissection was performed, and the renal artery was blocked 19 min during the operation. The tumor was completely removed as well as part of the normal renal tissue 1 cm from the edge. The operation ended smoothly.

## Results

**FIGURE 4:**
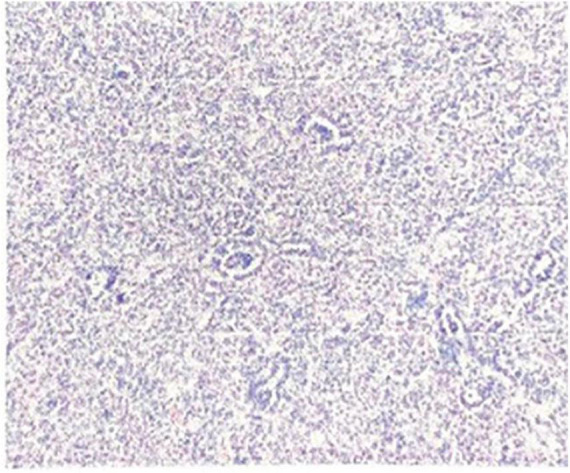
Microscopic appearance of the metanephric adenoma (HE 10 × 10).

Postoperative pathology: The tumor was oval in shape, about 58 × 35 × 34 mm in size, with a small amount of kidney tissue locally attached on the surface. The cut surface was gray and white with focal gray red, solid and medium in quality. There were several palpable nodules in perirenal fat sacs and lymph nodes, with a size of 3 × 2 × 2 mm to 10 × 6 × 2 mm. Microscopic observation: the tumor tissue was a lamellar, small tubular, and focal glomerulus-like structure. The tumor cells were uniform in size, with hyperchromatic nuclei and rare mitotic signs. No necrosis was observed (Figure 4). Immunohistochemical results: Vim(+), CK(multifocal+), WT1(+), Pax2(+), CD10(−), Desmin(−), Myogenin(−), MyoD1(−), S100(−), SALL4(−), Syn(−), CgA(−), CD99(+), INI-1(+), P53(local weak+), and Ki67(hot spot about 10%+). No lymph node metastasis was observed. Molecular detection by ARMS fluorescence quantitative PCR: BRAF mutation V600E (ABIViia7 fluorescence quantitative PCR instrument, Xiamen Ed human BrafV600E gene mutation detection kit). Immunohistochemical results of consultation in Children’s Hospital of Fudan University: PAX8(+), CD56(+), CD57(+), Cadherin17(−), P504S a few(+), CK7(−), P16 a few (+), WT1(+), pan-TRK(−), CD99a few(+), Ki-67(+)3%, and PNL2(−). Pathological diagnosis: MA.

## Discussion

Brisigotti et al. (9) researched more than 100 cases of pediatric renal tumors and identified a unique case in 1992; the tumor cells were well differentiated and morphologically benign, with no mitotic appearance and adenoma shape. They named MA for the first time and thought it might represent the benign counterpart of nephroblastoma. At the 1996 Heidelberg conference on “The influence of molecular genetics on the classification of renal cell neoplasms,” renal cell neoplasms were further divided into MA, mephirenal adenofibroma, papillary renal adenoma, and renal eosinoma (10). Posterior renal tumor was added to the WHO renal tumor classification in 2004, which included MA, adenofibroma of the posterior kidney, and mesenchymal tumors of the posterior kidney (11).

Metanephric adenoma occurs at all ages and has been reported from 15 months to 83 years old. Its clinical manifestations are not specific; most patients are only occasionally found during physical examination (12). In 1995, the Pathology Institute of Washington, United States, reported the largest number of MA studies to date; they found that 22% of patients had lower back pain, 10% had hematuria, and 10% had significant abdominal masses (13). Among these clinical manifestations, the incidence of polycythemia is relatively high, and some studies believe that MA can lead to polycythemia in patients by producing erythropoietin and some cytokines (2, 14).

Metanephric adenoma lacks specificity in imaging. Ultrasound usually presents as a well-defined round solid mass with few vessels and may be hyperechoic, isechoic, or hypoechoic (15). CT plain scan shows well-defined lesions that are heterogeneous as the tumor grows and may have small spotty calcifications. The mass may be enlarged after contrast-enhanced scan, but to a lesser degree than normal kidney, and is usually accompanied by hemorrhage and necrosis (16). MRI plain scan shows low or isosignal lesions on T1- and T2-weighted images, while the degree of enhancement of renal parenchyma decreased on enhanced MRI, and diffusion weighted magnetic resonance imaging (DWI) showed obvious high signal (17).

With the increase of MA case reports, the research of immunohistochemistry, molecular biology, and genetics has been gradually deepened. MA can occur in any part of the kidney, usually unilateral, with or without capsule, and some may be accompanied by cystic changes, hemorrhage, or necrosis. Histologically, MA can appear as spherical masses and bud-like structures. These two special forms are unique organizational structures of MA and have diagnostic value. Immunohistochemical staining of CK7 negative, AMACR negative, WT1 positive and CD57 positive were considered as the characteristics of MA; Kinney (18) found this feature in most MA cases (31/37) and suggested that only different immunostaining patterns are present and further FISH analysis is recommended. Cadherin 17 (CDH17) is mainly expressed in normal intestinal and digestive tract tumors. Yakirevich et al. (19) first suggested that CDH17 was a sensitive (81%) and highly specific (100%) marker of MA. Choueiri et al. (20) identified BRAF V600E mutations in 26 of 29 MA cases, providing the first evidence that BRAF V600E mutations are present in 90% of MA cases and can be used as a potential diagnostic tool. Udager et al. (21) showed that BRAF V66E had a sensitivity of 88% and specificity of 100%.

In pediatric cases, MA and nephroblastoma are difficult to identify, and fine needle biopsy is still controversial (13, 22, 23). Barroca et al. (24) believes that immunohistochemical and molecular studies are still needed to confirm the diagnosis of MA even if the cellular needle biopsy specimen suggests a diagnosis of MA. Since MA is considered by most scholars to be a benign lesion, the surgical method is gradually changing from nephrectomy to nephron-sparing surgery (5, 25, 26). Although most scholars consider MA to be a benign lesion, cases with metastasis or with malignant changes have been reported (27–29). Therefore, long-term active monitoring and follow-up are still needed after surgery.

In this case, stage I nephroblastoma was considered in preoperative examination, and nephron sparing surgery was planned (30). Preoperative chemotherapy was performed according to CCCG-WT-2016 (8). CT review after chemotherapy indicated that the tumor had shrunk by about 1 cm, and postoperative pathological examination showed no manifestations of tumor cells such as lysis and necrosis. To our knowledge, this is the first case of MA in children who received chemotherapy drugs before surgery. Whether the tumor shrinkage on imaging was caused by chemotherapy drugs remains to be further researched.

## Conclusion

Metanephric adenoma is mostly reported as individual cases at present, and the incidence rate of pediatrics is lower. There is a lack of medical evidence in a large number of cases. We considered that the clinical manifestations and imaging examinations of children with MA lack specificity, and partial nephrectomy with nephron sparing is the preferred treatment for children with MA. Long-term follow-up data and in-depth molecular genetic studies are still needed to determine the benign and malignant effects of MA and whether chemotherapy drugs have an impact on it.

## Data Availability Statement

The original contributions presented in the study are included in the article/supplementary material, further inquiries can be directed to the corresponding author.

## Ethics Statement

Written informed consent was obtained from the minor(s)’ legal guardian/next of kin for the publication of any potentially identifiable images or data included in this article.

## Author Contributions

SH wrote the manuscript. ZW and WB enrolled the information of the child. SC collected the imaging and pathological information. ZZ and YL supported the project. All authors reviewed the manuscript.

## Conflict of Interest

The authors declare that the research was conducted in the absence of any commercial or financial relationships that could be construed as a potential conflict of interest.

## Publisher’s Note

All claims expressed in this article are solely those of the authors and do not necessarily represent those of their affiliated organizations, or those of the publisher, the editors and the reviewers. Any product that may be evaluated in this article, or claim that may be made by its manufacturer, is not guaranteed or endorsed by the publisher.
